# Genetic Variation in ADAMTS13 is Related to VWF Levels, Atrial Fibrillation and Cerebral Ischemic Events

**DOI:** 10.1177/10760296221141893

**Published:** 2022-12-06

**Authors:** Ellen M. K. Warlo, Vibeke Bratseth, Alf-Åge R. Pettersen, Pål Andre Holme, Harald Arnesen, Ingebjørg Seljeflot, Trine B. Opstad

**Affiliations:** 1 Center for Clinical Heart Research, Department of Cardiology, 155272 Oslo University Hospital, Oslo, Norway; 2 56633 Institute of Clinical Medicine, 242998 University of Oslo, Oslo, Norway; 3 Department of Medicine, Vestre Viken HF, 72992 Ringerike Hospital, Hønefoss, Norway; 4 Department of Haematology, 155272 Oslo University Hospital, Oslo, Norway

**Keywords:** von Willebrand factor, polymorphisms, adamts13, cardiovascular disease, atrial fibrillation

## Abstract

**Introduction:**

ADAMTS13 cleaves von Willebrand factor (VWF) multimers into less active fragments. Both markers have been related to cardiovascular disease (CVD). We aimed to investigate the influence of ADAMTS13 single nucleotide polymorphisms (SNPs) on levels of ADAMTS13 and VWF, and CVD.

**Methods:**

The c.1342C>G, g.41635A>G and c.2699C>T polymorphisms were determined in patients with chronic coronary syndrome (n = 1000). VWF and ADAMTS13 were analyzed. Clinical endpoints after 2 years (n = 106) were unstable angina pectoris, myocardial infarction, non-hemorrhagic stroke and death.

**Results:**

The SNPs did not affect ADAMTS13 levels. The 41635A-allele associated with higher VWF levels (*P* < .001). Patients with the 1342G-allele had significantly higher frequency of previous atrial fibrillation (n = 26, *P* = .016) and cerebral ischemic events (n = 47, *P* = .030). Heterozygous of the 1342CG variant experienced more clinical endpoints compared to homozygous (CC and GG) (*P* = .028).

**Conclusion:**

The association between the 41635A-allele and VWF indicates a role for this polymorphism in VWF regulation. ADAMTS13 has previously been linked to atrial fibrillation and ischemic stroke, and our findings suggest that the 1342G-allele may be of significance. The association between the 1342CG genotype and endpoints needs further investigations.

Clinicaltrials.gov, ASCET, NCT00222261. https://clinicaltrials.gov/ct2/show/NCT00222261?term=NCT00222261&draw=2&rank=1

## Background

ADAMTS13 (A disintegrin and metalloproteinase with thrombospondin type 1 motif, member 13) is an enzyme cleaving ultra large von Willebrand factor (VWF) multimers into smaller fragments, thereby reducing VWFs prothrombotic properties.^1^ ADAMTS13 and VWF are important proteins involved in the delicate balance between thrombosis and bleeding. An imbalance in circulating levels of these proteins may lead to life threatening disorders as seen with thrombotic thrombocytopenic purpura (TTP).^2^

VWF is a well-established marker of endothelial activation and elevated levels have been associated with an increased risk of cardiovascular disease (CVD).^3^ ADAMTS13 has also been related to CVD, both independently and combined with VWF, but the results are diverging and more prospective studies are needed.^2,4,5^

Genetics are responsible for a considerable part of the variation in VWF levels and function, as seen in von Willebrand disease.^6^ Less clear is how genetic variation affects ADAMTS13. Some single nucleotide polymorphisms (SNPs) have been reported to influence ADAMTS13 levels and activity, but more research is necessary.^7,8^ In the present study, we selected three SNPs that have been associated with CVD, but are limitedly explored in patients with stable coronary artery disease (CAD). Other polymorphisms were considered, but excluded as the minor allele frequency (MAF) were too low to be investigated in this dataset. Both exon and intron variants were chosen as SNPs located in exons can lead to amino acid changes and influence protein activity, and intron variants may affect gene regulation and expression.

The c.1342C>G, g.41635A>G and c.2699C>T polymorphisms were investigated in this study. The c.1342C>G (rs2301612) variant is located in exon 12 in the cysteine rich domain of the ADAMTS13 gene, leading to an amino acid change from glutamine to glutamic acid at position 448 (Q448E). The SNP has been associated with a reduced risk of ischemic stroke.^9^ The variant g.41635A>G (rs4962153), located in intron 28, has been associated with increased risk of ischemic stroke,^9^ reduced ADAMTS13 antigen and activity levels^8^ and increased VWF antigen levels.^10^ The variant c.2699C>T (rs685523) is located in exon 21 in one of the thrombospondin type 1 repeats, resulting in a missense mutation at position 900 with the amino acid shift from alanine to valine (A900V). This variant has been associated with increased risk of death in a CAD population, and has also been related to type 2 diabetes (T2DM).^11,12^ Beyond these results, the importance of these SNPs on ADAMTS13 and VWF levels, as well as CVD is limited investigated. To our knowledge, no prospective study has investigated the effects of these SNPs on circulating ADAMTS13 and VWF levels and simultaneously the onset of future cardiovascular events in CAD patients.

Our aim was to investigate whether the selected ADAMTS13 genetic variants were related to levels of ADAMTS13 and VWF in a CAD population, and to explore any associations to CVD subgroups and future cardiovascular events. Our hypothesis was that these variants would influence ADAMTS13, with subsequent effects on VWF levels and the severity of CVD.

## Materials and Methods

### Study Population

The present investigation is an observational study, and a substudy of the ASCET trial (“Aspirin Nonresponsiveness and Clopidogrel Endpoint Trial”) performed at Center for Clinical Heart Research, Oslo University Hospital, Ullevaal, Oslo.^13^ The ASCET study is a randomized controlled clinical trial including 1001 patients with angiographically verified chronic coronary syndrome (CCS). Patients were enrolled between March 2003 and July 2008. All patients were on single antiplatelet therapy with aspirin prior to randomization. Individuals using oral anticoagulants were excluded. At inclusion, patients were randomized to either continue with aspirin 160 mg/d or change to clopidogrel 75 mg/d. They were followed for 2 years and clinical endpoints were registered. The primary endpoints include unstable angina pectoris (UAP), myocardial infarction (MI), non-haemorrhagic stroke and all-cause mortality. The study is approved by the regional ethics committee and all patients gave their written consent. The study is registered at https://www.clinicaltrials.gov/ (identification No. NCT00222261).

### Clinical Subgroups at Baseline

History of MI, atrial fibrillation (AF), ischemic stroke and transient ischemic attacks (TIA) were registered from the patients’ medical files. Diabetes includes patients with treated type 1 diabetes mellitus (T1DM) or T2DM, or fasting blood glucose > 7 mmol/L. Hypertensives were defined as patients with treated hypertension. Current smokers includes patients still smoking or former smokers who had quit less than 3 months prior to inclusion. Cerebral ischemia was defined as a composite of ischemic stroke and TIA, not including cerebral hemorrhage.

### Blood Sampling

Blood samples were collected at inclusion between 08.00 and 10.30 AM under fasting conditions without morning medication. Ethylenediamine tetraacetic acid (EDTA) whole blood was kept frozen until DNA extraction. Citrated blood (0.129 M in dilution 1:10) was stored on ice and separated within 30 min by centrifugation at 4 °C and 3000 × g for 20 min for plasma preparation. Routine analyses were performed with conventional laboratory methods. All materials were stored at −80 °C until further analysis. We have previously published the results from the VWF and ADAMTS13 measurements used in this study,^13,14^ which were analyzed in citrated plasma using Asserachrom^®^ VWF Ag (Stago Diagnostica, Asnieres, France), IMUBIND^®^ (Sekisui Diagnostics GmbH, Pfungstadt, Germany) and TECHNOZYM^®^ ADAMTS-13 activity (Technoclone, Vienna, Austria), respectively. Inter-assay coefficients of variation for the analyses were 4.8, 8.7, 10.1%, respectively.

### Genotyping

DNA was extracted from EDTA blood by the use of the MagNA Pure LC DNA Isolation kit on the MagNA Pure LC Instrument (Roche diagnostics, Germany). DNA purity and quantity were tested on the NanoDrop, ND-1000 (Saveen Werner, Sweden). Genotyping of the c.1342C>G (rs2301612), g.41635A>G (rs4962153) and c.2699C>T (rs685523) polymorphisms were performed with the following TaqMan^TM^ SNP Genotyping Assays: ID C_11571465_1, C_32355793_10 and C_998032_10, respectively (Applied Biosystems, Foster City, CA, USA). For assay validation, and as some samples were non-detectable or failed to discriminate between the different alleles, 1–4% of the analyses were repeated. There were no discrepancies in the detected genotypes between the repeated runs. DNA was not available or not detectable in less than 1% of the samples, thereof a discrepancy in numbers of AF and cerebral ischemic events is present between Table 1 and Table 2/Supplementary Table S1.

**Table 1.** Clinical Characteristics at Baseline.

	Total Population (n = 1000)
Age (years)^a^	62.4 (36-81)
Sex, female, n (%)^d^	218 (21.8)
Race, white, n (%)^d^	968 (96.8)
* Cardiovascular risk factors, n (%)* ^d^	
Current smoking	203 (20.3)
Hypertension	556 (55.7)
Diabetes mellitus	200 (20.0)
Atrial fibrillation	27 (2.7)
Previous myocardial infarction	436 (43.7)
Previous percutaneous coronary intervention	379 (38.0)
Previous coronary artery bypass grafting	185 (18.5)
Previous cerebral ischemia^e^	48 (4.8)
Systolic blood pressure, mm Hg^b^	140 ± 19
Diastolic blood pressure, mm Hg^b^	82 ± 10
Body mass index, kg/m^b^	27.4 ± 3.7
* Biochemical analyses*	
Total cholesterol, mmol/L^b^	4.55 ± 0.98
LDL cholesterol, mmol/L^b^	2.53 ± 0.83
HDL cholesterol, mmol/L^b^	1.33 ± 0.41
Triglycerides, mmol/L^c^	1.31 (0.93, 1.84)
VWF antigen, IU/mL^c^	1.05 (0.82, 1.33)
ADAMTS13 antigen, ng/mL^c^	532 (461, 606)
ADAMTS13 activity, IU/mL^c^	1.03 (0.83, 1.19)
Ratio VWF/ADAMTS13 antigen, × 10^−3^ IU/ng ^cf^	1.98 (1.51, 2.63)
Ratio VWF/ADAMTS13 activity, IU/IU ^c^	1.07 (0.77, 1.52)
* Medication, n (%)* ^d^	
Statins	982 (98.3)
B-blockers	755 (75.8)
Calcium channel blockers	255 (25.6)
ACE-inhibitors	263 (26.5)
ARBs	239 (24.0)

Abbreviations: ACE, angiotensin-converting enzyme; ARBs, angiotensin II receptor blockers; LDL, low-density lipoprotein; HDL, high-density lipoprotein.

^a^ Mean (range), ^b^Mean ± SD, ^c^Median (25^th^, 75^th^ percentiles), ^d^valid percent, ^e^Cerebral ischemia includes ischemic stroke and TIA (transient ischemic attack) ^f^For convenience, the value is presented in 10^−3^. The low value is due to different units in the ratio.

**Table 2.** Genotype Distribution of the Different ADAMTS13 SNPs According to CAD Subgroups at Baseline.

		Sex (Female)		Diabetes		Hypertension		AF		Cerebral Ischemia^a^		MI	
		+	-	+	-	+	-	+	-	+	-	+	-
c.1342C>G	CC	62	225	58	229	160	127	2	285	7	280	126	161
	CG	115	374	86	403	268	221	15	473	30	459	213	274
	GG	41	176	53	164	126	90	9	208	10	206	93	124
	p^1^		.39		.11		.69		**.038**		.07		.97
	p^2^		.87		.85		.97		**.016**		**.030**		.90
g.41635A>G	GG	151	541	144	548	390	301	19	673	38	635	310	382
	GA	61	209	47	223	149	121	8	261	8	262	109	159
	AA	6	27	8	25	17	16	0	33	1	32	15	18
	p^1^		.84		.41		.82		.61		.22		.50
	p^2^		.92		.34		.63		.93		.08		.29
c.2699C>T	CC	182	608	153	637	447	342	22	767	34	755	330	458
	CT	33	156	44	145	97	92	4	185	14	175	94	95
	TT	2	12	3	11	10	4	0	14	0	14	6	8
	p^1^		.20		.48		.21		.72		.14		.15
	p^2^		.08		.23		.31		.52		.13		.06

Abbreviations: AF, Atrial fibrillation; MI, myocardial infarction. ^a^Cerebral ischemia includes ischemic stroke and TIA (transient ischemic attack). p^1^ *P*-value represents difference in numbers of subject with the genotypes in different subgroups (Chi-squared test). p^2^ *P*-value represents difference in numbers of subjects with the genotypes in heterozygous and homozygous combined compared to the wild type (Chi-squared test). Significant *P*-values are highlighted with boldface. The number of patients in each group may differ slightly between the SNPs, as DNA was not available or the SNPs were not detectable in some samples.

### Statistical Analysis

Categorical variables are presented as numbers or percentages. Continuous variables are presented as mean ± SD in normally distributed variables and median (25^th^, 75^th^ percentiles) in skewed variables. Pearson's chi-squared test was used to test for deviation from the Hardy-Weinberg Equilibrium (HWE) and for group comparisons of categorical data. Group comparisons of continuous data were performed by using Mann-Whitney U test or Kruskal-Wallis test when appropriate. A *P*-value < .05 was considered statistically significant. SPSS versions 22-26 (SPSS Inc., IL, USA) have been used.

## Results

Baseline characteristics of the ASCET population^13^ are shown in Table 1. Results are given for 1000 patients, as blood samples from one patient were not available. All patients had angiographically verified CCS, mean age was 62 years and 22% were females. VWF and ADAMTS13 antigen were analyzed in all, whereas the ADAMTS13 activity analysis was successful in 955 patients.

Table 3 presents genotype frequencies for the investigated ADAMTS13 genetic variants. The MAF for the c.1342C>G, g.41635A>G and c.2699C>T SNPs was 0.47, 0.17 and 0.11, respectively. No deviation from the HWE was observed (*P* > .5 for all), and the genotype frequencies are in line with previous reports.^9,15^

**Table 3.** Genotype Frequencies of the Investigated ADAMTS13 SNPs.

Polymorphism	Genotype	Frequency
c.1342C>G	CC	287
	CG	489
	GG	217
	Total	993
	MAF	0.47
g.41635A>G	GG	692
	GA	270
	AA	33
	Total	995
	MAF	0.17
c.2699C>T	CC	790
	CT	189
	TT	14
	Total	993
	MAF	0.11

Abbreviation: MAF, Minor allele frequency. The total number of patients differs slightly between the SNPs, as DNA was not available or the SNPs were not detectable in some samples.

### ADAMTS13 Genotypes and Levels of ADAMTS13 and VWF

In Table 4, levels of ADAMTS13 antigen, ADAMTS13 activity, VWF antigen and VWF/ADAMTS13 ratios are shown according to ADAMTS13 genotypes. There were no significant differences in ADAMTS13 antigen or activity levels for any of the investigated polymorphisms. A tendency of higher ADAMTS13 antigen levels was observed with increasing number of the 1342 G-allele (*P* = .07). The g.41635A>G polymorphism was associated with higher VWF levels, with significant differences between genotypes and in A-allele carriers compared to the wild type (*P* < .001, both). The VWF/ADAMTS13 antigen ratio was also significantly higher in A-allele carriers (*P* < .001).

**Table 4.** Levels of ADAMTS13 and VWF According to ADAMTS13 SNPs and Their Respective Genotypes.

		ADAMTS13 Antigen (ng/mL)	ADAMTS13 Activity (IU/mL)	VWF Antigen (IU/mL)	VWF/ADAMTS13 Antigen (x10^−3^ IU/ng)^a^	VWF/ADAMTS13 Activity (IU/IU)
c.1342C>G	CC	526 (453, 589)	1.04 (0.83, 1.21)	1.05 (0.81, 1.35)	2.02 (1.52, 2.68)	1.08 (0.76, 1.45)
	CG	531 (461, 599)	1.02 (0.84, 1.16)	1.06 (0.84, 1.33)	2.00 (1.54, 2.65)	1.07 (0.77, 1.54)
	GG	539 (472, 633)	1.03 (0.80, 1.20)	1.05 (0.83, 1.32)	1.91 (1.40, 2.66)	1.06 (0.75, 1.53)
	p^1^	.07	.47	.95	.49	.83
	p^2^	.31	.22	.76	.98	.57
g.41635A>G	GG	533 (461, 613)	1.02 (0.80, 1.18)	1.02 (0.78, 1.29)	1.90 (1.41, 2.53)	1.04 (0.74, 1.50)
	GA	533 (462, 591)	1.04 (0.86, 1.20)	1.15 (0.92, 1.41)	2.19 (1.71, 2.84)	1.10 (0.85, 1.55)
	AA	480 (450, 583)	1.03 (0.86, 1.14)	1.18 (1.06, 1.38)	2.45 (1.66, 3.07)	1.13 (0.97, 1.58)
	p^1^	.27	.27	**<.001**	**<.001**	.12
	p^2^	.42	.12	**<.001**	**<.001**	.05
c.2699C>T	CC	533 (463, 605)	1.04 (0.84, 1.19)	1.07 (0.84, 1.34)	2.02 (1.53, 2.68)	1.07 (0.77, 1.53)
	CT	521 (447, 608)	1.01 (0.81, 1.19)	1.02 (0.80, 1.30)	1.91 (1.46, 2.56)	1.06 (0.76, 1.38)
	TT	535 (474, 661)	0.96 (0.48, 1.17)	0.96 (0.78, 1.06)	1.53 (1.42, 2.15)	0.91 (0.76, 4.35)
	p^1^	.37	.41	.15	.16	.84
	p^2^	.38	.22	.10	.18	.56

p^1^ *P*-value represents difference in circulating levels of the markers between ADAMTS13 genotypes (Kruskal-Wallis test). p^2^ *P*-value represents difference in circulating levels of the markers in heterozygous and homozygous combined compared to the wild type (Mann-Whitney U test). Significant *P*-values are highlighted with boldface. ^a^For convenience, the value is presented in 10^−3^. The low value is due to different units in the ratio.

### ADAMTS13 Genotype Distribution in Clinical Subgroups at Baseline

**Figure 1.**
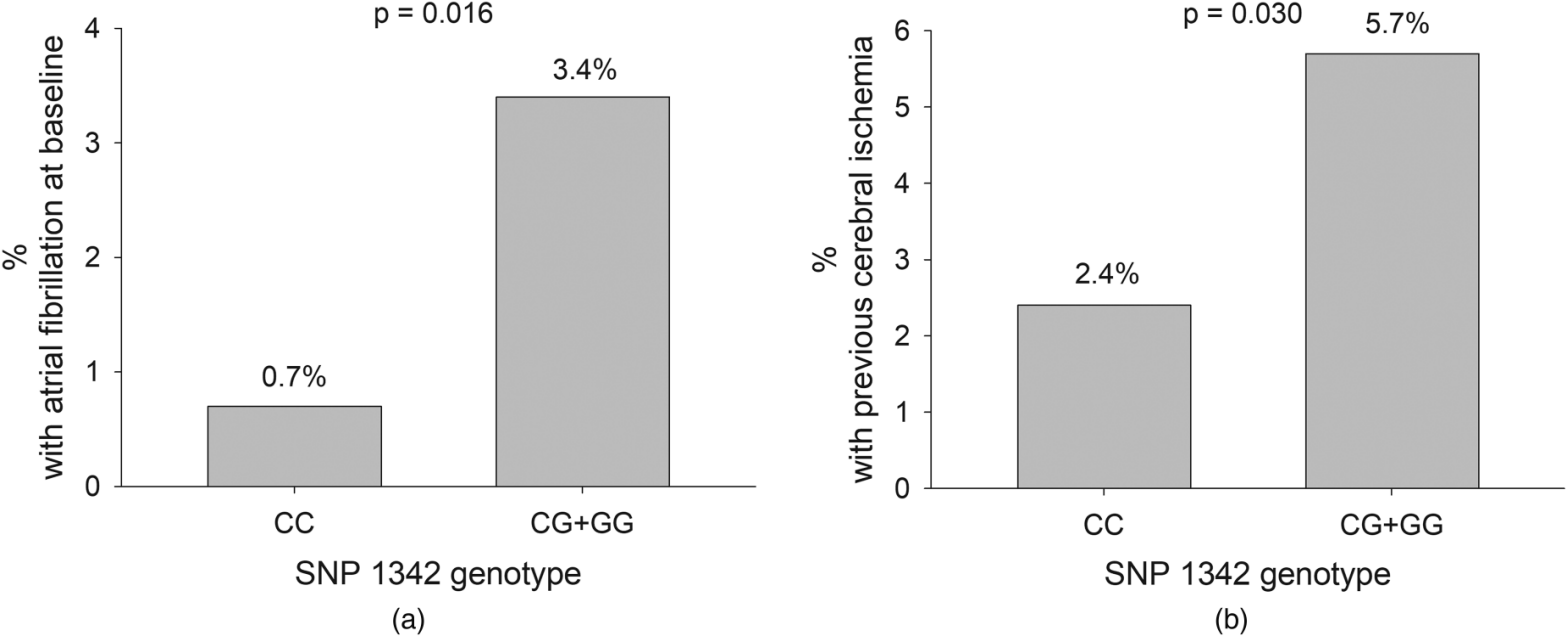
(a) The frequency of AF at baseline according to ADAMTS13 c.1342C>G SNP. (b) The frequency of previous cerebral ischemic events at baseline according to ADAMTS13 c.1342C>G SNP.

There were no significant differences in genotype distribution with respect to sex, diabetes, hypertension or MI, as shown in Table 2. Patients with the 1342 G-allele presented with significantly higher frequency of AF (*P* = .016) and previous cerebral ischemic events (*P* = .030) (Figure 1), still significant after adjusting for age and sex (*P* = .028 and *P* = .032, respectively). The occurrence of AF (n = 26) according to c.1342C>G genotypes were: CC 0.7%, CG 3.1% and GG 4.1% and for cerebral ischemic events (n = 47): CC 2.4%, CG 6.1% and GG 4.6%. There were limited overlap between the two groups, with only one patient presenting with both a history of AF and cerebral ischemic event. In Supplementary Table S1 the cerebral ischemic events are stratified according to TIA (n = 23) and ischemic stroke (n = 26). The 1342 G-allele was significantly associated with TIA (*P* = .009), but no tendency or significance was found for ischemic stroke analyzed separately (*P* = .51). The occurrence of TIA according to c.1342C>G genotypes were: CC 0.3%, CG 3.3%, GG 2.8% and for ischemic stroke: CC 2.1%, CG 3.3%, GG 1.9%.

### Associations Between ADAMTS13 Genotypes and Clinical Endpoints

After 2 years follow-up, 106 clinical endpoints (UAP, MI, non-haemorrhagic stroke or death) were recorded. In Table 5, the number of composite endpoints are presented according to the ADAMTS13 genotypes. There was a significant difference in number of endpoints between ADAMTS13 c.1342C>G genotypes (*P* = .028) with the following frequencies: CC 8.4%, CG 13.1% and GG 7.4%. The significance was lost when pooling heterozygous and homozygous for the minor allele (*P* = .17). Due to the previously reported association between the c.1342C>G SNP and ischemic stroke,^9^ data were analyzed separately for non-hemorrhagic stroke, however, without any significant association. Moreover, no significant associations between the primary endpoints and the ADAMTS13 g.41635A>G or c.2699C>T polymorphisms were observed. The baseline characteristics of patients with and without clinical endpoints were similar, except for a higher frequency of previous MI and coronary artery bypass surgery in patients with clinical endpoints, as shown in Supplementary Table S2.

**Table 5.** The Number of Composite Endpoints According to the Different ADAMTS13 SNPs.

		Composite Endpoints	
		+	-
c.1342C>G	CC	24	263
	CG	64	425
	GG	16	201
	p^1^	**.028**	
	p^2^	.17	
g.41635A>G	GG	69	623
	GA	31	239
	AA	6	27
	p^1^	.29	
	p^2^	.29	
c.2699C>T	CC	81	709
	CT	21	168
	TT	2	12
	p^1^	.84	
	p^2^	.66	

p^1^ *P*-value represents difference in numbers of subject with the genotypes in different subgroups (Chi-squared test). p^2^ *P*-value represents difference in numbers of subjects with the genotypes in heterozygous and homozygous combined compared to the wild type (Chi-squared test). Significant *P*-values are highlighted with boldface. The number of endpoints differs slightly among the SNPs, as DNA was not available or the SNPs were not detectable in some samples.

## Discussion

The main findings in our study in patients with CCS were that the ADAMTS13 41635 A-allele associated with higher VWF levels and a higher VWF/ADAMTS13 antigen ratio. Patients with the ADAMTS13 1342 G-allele presented with higher frequency of AF and cerebral ischemic events at baseline, and a tendency towards higher clinical endpoint rate after two years in the heterozygous group. We found no significant associations between the investigated polymorphisms and levels of ADAMTS13 antigen or activity. The clinical impact of each polymorphism is discussed separately.

### SNP c.1342C>G

Although no significant associations with levels of ADAMTS13 antigen, activity or VWF antigen were found, a tendency of increasing ADAMTS13 antigen levels was observed for the G-allele, as previously reported in healthy individuals,^7^ but contradictory reported by others in subjects with CVD.^16^ Our results on the polymorphisms’ impact on ADAMTS13 are in accordance with previous reports.^10,15,17^

We observed higher occurrence of previous cerebral ischemic events at baseline in patients with the 1342 G-allele. Analyzed separately, the association was prominent for TIA, while no significant association was observed for ischemic stroke. The number of G-allele carriers were low in both groups, which may explain the lack of significance in stroke patients. These results are partly in contrast to Hanson *et al* who found the allele to be non-significantly associated with a decreased risk of ischemic stroke.^9^ Lower levels of ADAMTS13 antigen and activity, as well as several other ADAMTS13 SNPs, have previously been associated with ischemic stroke.^5,15,18^ In the present study, patients with the G-allele also presented with an increased occurrence of AF at baseline, not previously reported. Low ADAMTS13 and high VWF levels have been associated with AF and left atrial remodeling.^19,20^ As the observed association of the ADAMTS13 c.1342C>G variant with previous cerebral ischemic events and AF was not accompanied by lower ADAMTS13 or higher VWF levels in our study, the mechanisms behind these observations are unclear. The sample size may be too small or it may represent a random association. The 1342C>G variant leads to a missense mutation (amino acid substitution from glutamine to glutamic acid) that may modify ADAMTS13 activity, however, not detected by the utilized methods and thereby increase the risk of disease.

It is well known that AF predisposes for cardiac embolisms that may cause a TIA or ischemic stroke. We therefore investigated whether there was any overlap between the groups, and only one patient presented with both AF and previous cerebral ischemic event. As the use of oral anticoagulation, highly recommended in patients with AF and previous stroke, was an exclusion criterion in our study, the group with combined AF and ischemic stroke is probably underrepresented. Our results may therefore indicate that subjects having this polymorphism have an independent increased risk of AF and cerebral ischemic events. On the other hand, one can hypothesize that the patients with previous ischemic events might have unrecognized paroxysmal AF.

The c.1342C>G SNP was not associated with sex, hypertension, MI or diabetes, in concordance with a previous report.^11^ There was a significant difference in number of clinical endpoints according to genotypes after 2 years, but with no clear trend. A higher endpoint rate in heterozygous compared to homozygous was observed, with similar endpoint rate in homozygous, ensuing a random or uncertain association of the variant allele with new clinical events. A prospective study in a population similar to ours, showed no increased risk for cardiovascular events or death.^11^

### SNP g.41635A>G

We observed no significant differences in ADAMTS13 antigen or activity when investigating the g.41635A>G intron variant. The results on ADAMTS13 activity are in line with previous reports,^10,15^ but lower ADAMTS13 antigen and activity in patients with the AA genotype compared to the G-allele has also been described.^8^ The number of AA homozygous was limited in our study (n = 33), and the AA genotype was only numerically associated with lower ADAMTS13 antigen levels.

The 41635 A-allele was significantly associated with higher VWF levels, in accordance with a previous report.^10^ Also, the ratio VWF/ADAMTS13 antigen associated significantly with the presence of this SNP. We have previously shown the ratio to be of importance and a better prognostic marker than VWF alone in CCS.^14^ The mechanism behind the observed association between this SNP and VWF is unclear, but it is known that intronic variants can lead to altered gene expression due to an altered splicing of the exons. It may be that these patients actually have lower ADAMTS13 levels that leads to higher VWF levels, but that the MAF was too low for the ADAMTS13 results to reach significance in this population.

No significant associations with clinical subgroups or clinical outcome were observed, partly in line with the literature.^10^ In contrast, Hanson *et al* observed an increased occurrence of A-allele carriers in a population with ischemic stroke (n = 600) compared with controls.^9^ The lack of association in our study may be due to the lower number of patients with cerebral ischemic events.

### SNP c.2699C>T

The c.2699C>T exon variant did not affect ADAMTS13 antigen, activity or VWF antigen levels significantly. This is in line with other reports showing no influence of this SNP on ADAMTS13 activity.^15,21^ To our knowledge, this is the first study investigating this SNP in regards to ADAMTS13 antigen and VWF antigen.

We also found no significant associations with comorbidity at baseline or to clinical outcome after 2 years. This is in correspondence with Lopez *et al* who found no association to TIA, stroke, MI or hypertension in a population with systemic lupus erythematosus. However, in another study, CAD patients carrying the T-allele had a significantly increased risk of death, especially due to cardiac causes.^11^ In our material, we did observe a borderline significant (*P* = .06) higher occurrence of previous MI at baseline in T-allele carriers, but no association to death or MI after 2 years. The low endpoint rate in our study with 36 MIs and 9 deaths, may be too low to detect any association.

## Study Limitations

This is a substudy of the ASCET trial. Hence, the study was not designed and powered for the present investigation. All analyses are post hoc analyses, and the observations should be interpreted with caution. Patients on anticoagulants were excluded at inclusion, which may have led to selection bias especially regarding patients with AF, TIA and ischemic stroke. One main limitation was that VWF activity was not measured, however, VWF antigen and activity values seem to be correlated.^22,23^ In addition, ABO blood group is believed to influence VWF levels, and there seems to be a modest linkage disequilibrium between the ADAMTS13 gene and that of the glycosyltransferase controlling the ABO blood group. Unfortunately, blood type was not available in this study. The investigated SNPs may also be in linkage disequilibrium with other ADAMTS13 genetic variants that may alter properties of the ADAMTS13 protein or modify disease risk. The low number of patients in subgroups, the limited follow-up period and the low number of endpoints may have been inadequate to detect potential associations, compromising statistical error Type II.

## Conclusion

Our results indicate that the selected ADAMTS13 genetic variants do not influence plasma levels of ADAMTS13 antigen or activity in patients with CCS. The 41635 A-allele associated slightly with increased VWF levels, suggesting a role for this polymorphism in VWF regulation. As ADAMTS13 previously has been linked to AF and ischemic stroke, our findings suggest that the 1342 G-allele may be of significance. The possible association observed between the c.1342C>G SNP and clinical outcome after 2 years needs further investigations.

## Supplemental Material

sj-docx-1-cat-10.1177_10760296221141893 - Supplemental material for Genetic Variation in ADAMTS13 is Related to VWF Levels, Atrial Fibrillation and Cerebral Ischemic Events Click here for additional data file. Supplemental material, sj-docx-1-cat-10.1177_10760296221141893 for Genetic Variation in ADAMTS13 is Related to VWF Levels, Atrial Fibrillation and Cerebral Ischemic Events by Ellen M. K. Warlo, MD, Vibeke Bratseth, MSc, PhD, Alf-Åge R. Pettersen, MD, PhD, Pål Andre Holme, MD, PhD, Harald Arnesen, MD, PhD, Ingebjørg Seljeflot, PhD, and Trine B. Opstad, MSc, PhD in Clinical and Applied Thrombosis/Hemostasis

## List of Abbreviations

ADAMTS13: = A Disintegrin And Metalloproteinase with ThromboSpondin type 1 motif, member 13
AF: = Atrial fibrillation
ASCET: = Aspirin Nonresponsiveness and Clopidogrel Endpoint Trial
CAD: = Coronary artery disease
CCS: = Chronic coronary syndrome
CVD: = Cardiovascular disease
MAF: = Minor allele frequency
MI: = Myocardial infarction
SD: = Standard deviation
SNP: = Single nucleotide polymorphism
T1DM: = Type 1 diabetes mellitus
T2DM: = Type 2 diabetes mellitus
TIA: = Transient ischemic attack
TTP: = Thrombotic thrombocytopenic purpura
UAP: = Unstable angina pectoris
VWF: = von Willebrand factor
