# Supplementary material for: Genetic Variation in ADAMTS13 is Related to VWF Levels, Atrial Fibrillation and Cerebral Ischemic Events
Source: Clin Appl Thromb Hemost. 2022 Dec 6;28:10760296221141893. doi: 10.1177/10760296221141893 (PMC9732802; doi:10.1177/10760296221141893)
Supplement: sj-docx-1-cat-10.1177_10760296221141893 - Supplemental material for Genetic Variation in ADAMTS13 is Related to VWF Levels, Atrial Fibrillation and Cerebral Ischemic Events [file sj-docx-1-cat-10.1177_10760296221141893.docx]

**Supplementary Table S1 The frequency of cerebral ischemic events at baseline stratified into TIA and ischemic stroke according to the ADAMTS13 SNP 1342 C>G**

|  |  | Cerebral ischemia^¶^ (n=47) | | TIA (n=23) | | Ischemic stroke (n=26) | |
| --- | --- | --- | --- | --- | --- | --- | --- |
|  |  | + | - | + | - | + | - |
| 1342 C>G | CC | 7 | 280 | 1 | 286 | 6 | 281 |
|  | CG | 30 | 459 | 16 | 473 | 16 | 473 |
|  | GG | 10 | 206 | 6 | 210 | 4 | 212 |
|  | p^1^ |  | 0.07 |  | **0.029** |  | 0.44 |
|  | p^2^ |  | **0.030** |  | **0.009** |  | 0.51 |

^¶^ Cerebral ischemia includes ischemic stroke and TIA (transient ischemic attack).
p^1^ p-value represents difference in numbers of subject with the actual genotypes in different subgroups (Chi-squared test). p^2^ p-value represents difference in numbers of subjects with the actual genotypes in heterozygous and homozygous combined compared to the wild type (Chi-squared test). Significant p-values are highlighted with boldface.

**Supplementary Table S2 Clinical characteristics at baseline according to clinical endpoint**

|  | Endpoint (n=106) | No endpoint (n=894) | p-value^††^ |
| --- | --- | --- | --- |
| Age (years)^*^ | 62.9 (44.0-80.3) | 62.3 (36.4-80.8) | 0.500 |
| Sex, female, n (%)^§^ | 23 (21.7) | 195 (21.8) | 0.979 |
| Race, white, n (%)^§^ | 101 (97.0) | 867 (95.3) | 0.348 |
| *Cardiovascular risk factors, n (%)*^§^ |  |  |  |
| Current smoking | 23 (21.9) | 180 (20.1) | 0.670 |
| Hypertension | 63 (59.4) | 493 (55.2) | 0.408 |
| Diabetes mellitus | 24 (22.6) | 176 (19.7) | 0.472 |
| Atrial fibrillation | 5 (4.7) | 22 (2.5) | 0.176 |
| Previous myocardial infarction | 57 (53.8) | 379 (42.5) | **0.027** |
| Previous percutaneous coronary intervention | 44 (41.9) | 335 (37.6) | 0.390 |
| Previous coronary artery bypass grafting | 35 (33.0) | 149 (16.7) | **<0.001** |
| Previous cerebral ischemia^¶^ | 8 (7.5) | 40 (4.5) | 0.163 |
| Systolic blood pressure, mm Hg^†^ | 139±19 | 140±19 | 0.844 |
| Diastolic blood pressure, mm Hg^†^ | 82±5 | 82±10 | 0.755 |
| Body mass index, kg/m^†^ | 27.4±4.0 | 27.4±3.7 | 0.908 |
| *Biochemical analyses* |  |  |  |
| Total cholesterol, mmol/L^†^ | 4.53±0.99 | 4.55±0.98 | 0.872 |
| LDL cholesterol, mmol/L^†^ | 2.51±0.75 | 2.53±0.84 | 0.752 |
| HDL cholesterol, mmol/L^†^ | 1.33±0.38 | 1.34±0.41 | 0.891 |
| Triglycerides, mmol/L^‡^ | 1.25 (1.01, 1.87) | 1.32 (0.93, 1.84) | 0.856 |
|  |  |  |  |
|  | Endpoint (n=106) | No endpoint (n=894) | p-value^††^ |
| VWF antigen, IU/mL^‡^ | 1.11 (0.89, 1.33) | 1.05 (0.81, 1.33) | 0.469 |
| ADAMTS13 antigen, ng/mL^‡^ | 518 (456, 579) | 533 (462, 607) | 0.375 |
| ADAMTS13 activity, IU/mL^‡^ | 1.04 (0.79, 1.17) | 1.02 (0.83, 1.19) | 0.868 |
| Ratio VWF/ADAMTS13 antigen, x 10^-3^ IU/ng ^‡**^ | 2.09 (1.57, 2.64) | 1.97 (1.50, 2.64) | 0.330 |
| Ratio VWF/ADAMTS13 activity, IU/IU ^‡^ | 1.08 (0.74, 1.60) | 1.07 (0.77, 1.50) | 0.748 |
| *Medication, n (%)*^§^ |  |  |  |
| Statins | 105 (99.1) | 877 (98.2) | 0.523 |
| B-blockers | 78 (74.3) | 677 (76.0) | 0.701 |
| Calcium channel blockers | 29 (27.4) | 226 (25.4) | 0.661 |
| ACE-inhibitors | 32 (30.5) | 231 (26.0) | 0.324 |
| ARBs | 27 (25.5) | 212 (23.9) | 0.397 |

ACE; angiotensin-converting enzyme, ARBs; angiotensin II receptor blockers

^*^Mean (range), ^†^Mean±SD, ^‡^Median (25^th^, 75^th^ percentiles), ^§^valid percent, ^¶^Cerebral ischemia includes ischemic stroke and TIA (transient ischemic attack). ^**^For convenience, the value is presented in 10^-3^. The low value is due to different units in the ratio.^††^ p-values refer to differences between the groups with clinical endpoint and no endpoint. Significant p-values are highlighted with boldface.
